# A phase I trial of ispinesib, a kinesin spindle protein inhibitor, with docetaxel in patients with advanced solid tumours

**DOI:** 10.1038/sj.bjc.6604264

**Published:** 2008-03-04

**Authors:** S P Blagden, L R Molife, A Seebaran, M Payne, A H M Reid, A S Protheroe, L S Vasist, D D Williams, C Bowen, S J Kathman, J P Hodge, M M Dar, J S de Bono, M R Middleton

**Affiliations:** 1 Royal Marsden Hospital and Institute of Cancer Research, Downs Road, Sutton, Surrey SM2 5PT, UK; 2 Medical Oncology Unit, Cancer Research UK, Oxford Radcliffe Hospitals NHS Trust, The Churchill Hospital, Old Road, Headington, Oxford OX3 7LJ, UK; 3 GlaxoSmithKline Research and Development and Clinical Pharmacokinetics, Clinical Pharmacology and Discovery Medical Oncology, Research Triangle Park, NC 27709, USA; 4 Section of Molecular Therapeutics, Division of Surgery, Department of Oncology, Oncology, Reproduction and Anaesthetics, Imperial College London, Hammersmith Hospital, Du Cane Road, London W12 0HS, UK

**Keywords:** docetaxel, kinesin spindle protein, mitotic kinesin, phase I, SB-715992

## Abstract

The aim of this study is to define the maximum tolerated dose (MTD), safety, pharmacokinetics (PKs) and efficacy of ispinesib (SB-715992) in combination with docetaxel. Patients with advanced solid tumours were treated with ispinesib (6–12 mg m^−2^) and docetaxel (50–75 mg m^−2^). Docetaxel was administered over 1 h followed by a 1-h infusion of ispinesib on day 1 of a 21-day schedule. At least three patients were treated at each dose level. Blood samples were collected during cycle 1 for PK analysis. Clinical response assessments were performed every two cycles using RECIST guidelines. Twenty-four patients were treated at four dose levels. Prolonged neutropaenia and febrile neutropaenia were dose limiting in six and two patients, respectively. The MTD was ispinesib 10 mg m^−2^ with docetaxel 60 mg m^−2^. Pharmacokinetic assessment demonstrated concentrations of ispinesib and docetaxel, consistent with published data from single agent studies of the drugs. Seven patients (six hormone refractory prostate cancer (HRPC), one renal cancer) had a best response of stable disease (⩾18 weeks). One patient with HRPC had a confirmed >50% prostatic-specific antigen decrease. The MTD for ispinesib and docetaxel was defined and the combination demonstrated an acceptable toxicity profile. Preliminary PK data suggest no interaction between ispinesib and docetaxel.

Mitotic kinesins are a subset of the kinesin enzyme super family, and they are involved in the establishment and function of the mitotic spindle as well as cell cycle progression (Wood *et al*, 2001). In contrast to tubulin, mitotic kinesins are preferentially expressed in proliferating cells, with specific activity during mitosis, and are thus an attractive molecular target for anticancer therapy (Sakowicz *et al*, 2004). Kinesin spindle protein (KSP, Eg5, kinesin-5) provides the propulsive forces required to separate centrosomes during prophase, enabling them to migrate to opposite poles and establish a functional bipolar spindle (Blangy *et al*, 1995). Kinesin spindle protein is greatly expressed in proliferating over non-proliferating cells and in tumour tissue relative to normal tissue (Hegde *et al*, 2003). In *in vitro* experiments, cells treated with the prototype KSP inhibitor, monastrol, displayed abnormal, monopolar spindles with chromosomes attached via microtubules to a single pole, resulting in deranged cell division, mitotic cell cycle arrest and apoptosis (Mayer *et al*, 1999; Cochran *et al*, 2005).

Ispinesib (SB-715992), a potent and selective small molecule inhibitor of KSP, functions by inhibiting KSP ATPase and is 40 000 times more selective for KSP compared to other kinesins (Johnson *et al*, 2002). In preclinical studies, ispinesib inhibited growth in a wide range of human and murine cell lines with IC_50_ values of 1.2–9.5 nM. Treatment of SKOV3 ovarian tumour cells *in vitro* with 20 nM of ispinesib, or a Colo 205 colon cancer murine xenograft model with 30 mg kg^−1^ of intraperitoneal ispinesib, caused mitotic arrest with cells demonstrating unseparated centrosomes and monopolar mitotic spindles. Tumour growth delay was observed in xenograft models of colon (HT29, Colo201, Colo205), non-small cell lung (Calu-3) and pancreatic (Panc-01) cancers.

Phase I studies have evaluated three schedules of ispinesib administered on days 1–3 every 21 days, day 1 every 21 days and days 1, 8 and 15 every 28 days (Chu *et al*, 2003, 2004; Burris *et al*, 2004). In all studies, the dose-limiting toxicity (DLT) was prolonged neutropaenia or febrile neutropaenia. Other toxicities were mild with no significant neurotoxicity observed. Phase II studies are either ongoing or have been completed in multiple tumour types (Miller *et al*, 2005; Shahin *et al*, 2007).

A 9% objective response rate was observed in patients with metastatic breast cancer who had relapsed or were refractory to prior anthracycline and taxane therapy (Miller *et al*, 2005). Ispinesib has been evaluated in combination with both carboplatin and capecitabine in two phase I studies of 28 and 24 patients, respectively. The maximum tolerated dose (MTD) in both studies has been reported (Jones *et al*, 2006; Rodon *et al*, 2006).

This phase I study combines ispinesib with docetaxel. Docetaxel, a member of the taxane family, has activity in breast cancer, non-small cell lung cancer (NSCLC) and hormone refractory prostate cancer (HRPC) (Chan *et al*, 1999; Nabholtz *et al*, 1999; Fossella *et al*, 2000, 2003; Shepherd *et al*, 2000; Tannock *et al*, 2004). It is currently approved in several indications:

As second-line monotherapy for locally advanced or metastatic breast cancer (Taxotere®, 2005).
In combination with doxorubicin and cyclophosphamide for the adjuvant treatment of node-positive early breast cancer (Taxotere®, 2005).
In locally advanced or metastatic NSCLC as first-line therapy in combination with cisplatin or as second-line monotherapy.
In advanced gastric and gastro-oesophageal junction adenocarcinoma, it is approved as first-line therapy in combination with cisplatin and fluorouracil.
In squamous cell carcinoma of the head and neck as induction treatment for inoperable locally advanced disease with cisplatin and fluorouracil.
In combination with prednisone for patients with HRPC (Taxotere®, 2005).

Docetaxel binds reversibly to the beta subunit of tubulin, promoting microtubule assembly and stability, thereby blocking the cell cycle in mitosis (Eisenhauer and Vermorken, 1998). Myelosuppression is the DLT of docetaxel and occurs in >75% of patients treated with 60 mg m^−2^ (Taxotere®, 2005). Peripheral sensory neuropathy is another prominent toxicity, thought to occur as a result of tubulin stabilisation disrupting the architecture of non-dividing neuronal cells. Both docetaxel and ispinesib can induce mitotic arrest and apoptotic cell death; however, in the MX-1 tumour mouse xenograft model, preclinical data demonstrated synergy when both agents were used concurrently (data on file at GlaxoSmithKline).

The primary objectives of this study were to determine the safety and MTDs of ispinesib and docetaxel in combination. Secondary objectives were to define the pharmacokinetic (PK) profiles of both agents and their efficacy.

## MATERIALS AND METHODS

Eligible patients were recruited from The Royal Marsden Hospital, Sutton, and the Churchill Hospital, Oxford. GlaxoSmithKline sponsored the study. The study obtained full ethical approval and was conducted in accordance with ICH-GCP guidelines.

### Patient eligibility

Patients with histologically confirmed locally advanced or metastatic solid tumours, refractory to conventional therapy or for which no standard therapy exists, were eligible provided they met the following criteria: age ⩾18; Eastern Co-operative Oncology Group (ECOG) performance status (PS) 0–1; life expectancy of ⩾12 weeks; adequate haematopoietic (absolute neutrophil count (ANC) ⩾1.5 × 10^9^ l^−1^, platelet count ⩾100 × 10^9^ l^−1^, haemoglobin ⩾9 g dl^−1^), hepatic (transaminases <2 × upper limit of normal (ULN) or <1.5 × ULN if alkaline phosphatase ⩾3 × ULN, bilirubin ⩽ULN) and renal function (creatinine clearance >40 ml min^−1^ (calculated by the Cockroft–Gault Formula)) (Drinka and Langer, 1989); and a negative pregnancy test for females of child-bearing potential.

Exclusion criteria included the following: pre-existing neuropathy ⩾grade 2; unstable or pre-existing major medical conditions; evidence of symptomatic or uncontrolled brain metastases or leptomeningeal disease; major surgery or any anticancer therapy within the previous 4 weeks; lactating females; and unwillingness to use barrier contraception throughout the trial.

### Screening and study procedures

A full medical history and physical examination including PS, baseline symptoms, adverse events, vital sign assessment, haematology, coagulation and clinical chemistry were performed at baseline and prior to each treatment. A pregnancy test for females was performed prior to study entry only. Twelve-lead electrocardiograms were performed at baseline, prior to cycle 1 and immediately following the end of ispinesib infusion on day 1, cycle 1. Clinical chemistry, haematology and adverse event assessments were performed at days 8 and 15 of each cycle. Tumour assessment by computed tomography or magnetic resonance imaging, as defined by the RECIST guidelines (Therasse *et al*, 2000), was performed within 21 days prior to first treatment and every two cycles thereafter until discontinuation from the study. Patients were withdrawn from the study in the event of progressive disease (PD), unacceptable toxicity, withdrawal of consent or at the physician's discretion.

### Drug administration

Ispinesib was supplied by GlaxoSmithKline as a clear colourless solution in 4, 5 or 10 ml vials containing a 1 mg ml^−1^ solution of drug. Docetaxel (Taxotere® Sanofi-Aventis) was diluted as per the prescribing information.

All patients were premedicated with dexamethasone 8 mg p.o. b.i.d. for 3 days starting at day 0. On day 1, docetaxel was first administered as a 1 h i.v. infusion, followed by ispinesib, in 250 ml of 5% glucose administered i.v. over 1 h. The regimen was repeated every 21 days. Post-treatment anti-emetics comprised of domperidone 20 mg p.o. q.d.s.

### Dose escalation, definition of DLT and MTD

The starting dose of ispinesib was 8 mg m^−2^ (Chu *et al*, 2004). The starting dose of docetaxel was 60 mg m^−2^, selected on the basis of the anticipated myelotoxic profile of the combination. The planned dose escalation schedule is shown in Table 1.

Patients were treated in cohorts of three. In the absence of DLT, dose escalation continued in successive cohorts of three patients when three patients at the preceding dose level had completed a 21-day cycle. If one of the three patients experienced a DLT, an additional three patients were recruited to the same dose level. If no further DLTs were observed, dose escalation proceeded. If a second DLT was seen in the cohort of six, the MTD was exceeded and the dose below this was determined as the MTD.

Toxicities were graded according to the National Cancer Institute Common Terminology Criteria for Adverse Events Version 3.0 (NCI-CTCAE v 3.0). Dose-limiting toxicity was defined as any of the following: ⩾grade 3 clinically significant non-haematological toxicity (excluding ⩾grade 3 nausea, vomiting or alopaecia), ⩾grade 3 nausea/vomiting/diarrhoea uncontrolled by aggressive therapy, grade 4 neutropaenia lasting ⩾5 days, grade 3 or 4 neutropaenia with fever ⩾38.5°C or infection, grade 4 thrombocytopaenia, inability to commence next cycle of treatment within 2 weeks of scheduled dosing due to an unresolved toxicity such that the original eligibility criteria were no longer met, grade 2 toxicity, which is considered a DLT by the investigator, ⩾grade 2 non-haematological toxicity that persisted beyond cycle 1 and was considered a DLT by the investigator.

### Dose modification

Each new cycle of treatment was administered only if the following criteria were met: ANC >1.5 × 10^9^ l^−1^, platelet count >100 × 10^9^ l^−1^ and recovery from all clinically significant, non-haematological toxicity (apart from alopaecia) to ⩽grade 1. Treatment could be delayed for up to 2 weeks to allow these criteria to be met, otherwise treatment was discontinued.

In the case of DLT, on recovery to re-treatment criteria, the doses of both ispinesib and docetaxel were reduced 1 dose level for the subsequent cycle (Table 1). In the case of grade 2 neuropathy, treatment was withheld until resolved to grade 1 and the dose of docetaxel alone was reduced by 1 dose level. Colony-stimulating factors were prohibited during cycle 1 of treatment, but could be used in cases of febrile neutropaenia in subsequent cycles.

### Pharmacokinetic sampling

Plasma PK sampling was carried out on day 1 of cycle 1. A 4 ml blood sample was taken at the following time points relative to the docetaxel infusion: prior to and 1, 2, 4–6 and 24 h after the end of docetaxel infusion. Further sampling for a pPK interaction was to be performed at the MTD, if this was found to be less than dose level +3 (Table 1).

Ispinesib and docetaxel were extracted from plasma samples and then analysed by HPLC-MS/MS using a TurboIonSpray™ interface and multiple reaction monitoring by the GlaxoSmithKline Division of Drug Metabolism and Pharmacokinetics.

## RESULTS

Twenty-four patients (21 male, 3 female), with a median age of 61.2 years, were treated between June 2004 and June 2005 (Table 2). A wide range of tumour types was treated, with the most common tumour type being HRPC.

### Dose-limiting toxicities

Table 3 summarises the number of patients treated, cycles administered and DLTs per dose level. Patients received a median of three cycles of treatment (range 1–10). Six patients received six or more cycles of treatment and 18 discontinued treatment prematurely due to PD (12), adverse events (4), physician choice (1) and patient choice (1). At dose level 0 (ispinesib 8 mg m^−2^ and docetaxel 60 mg m^−2^), a patient with HRPC experienced a DLT of prolonged grade 4 neutropaenia during course 1. The cohort was thus expanded to six patients. There were no further DLTs.

At 8 mg m^−2^ ispinesib and docetaxel 75 mg m^−2^, after an initial patient with colorectal adenocarcinoma experienced a DLT of prolonged grade 4 neutropaenia, the cohort was expanded to a total of six patients. The second patient experienced prolonged grade 4 neutropaenia with fever. In order to further clarify the MTD, dose level A, at 10 mg m^−2^ ispinesib and docetaxel 60 mg m^−2^, was evaluated. There were no DLTs in the three patients treated at this dose level. In view of recurrent prolonged neutropaenia, we modified the dose escalation procedure to maintain the dose of docetaxel at 60 mg m^−2^ and increase the dose of ispinesib only.

In cohort A1, ispinesib was administered at 12 mg m^−2^ and docetaxel at 60 mg m^−2^. After a patient with renal carcinoma developed prolonged grade 4 neutropaenia, the cohort was expanded to six patients. A further two patients – one with duodenal carcinoma and a second with squamous cell carcinoma of the cervix – experienced prolonged grade 4 neutropaenia. With three out of six patients at this dose level experiencing DLT, the MTD was defined as ispinesib 10 mg m^−2^ and docetaxel 60 mg m^−2^. The MTD cohort was expanded by a further three patients with no further DLTs.

### Haematological toxicity

All patients were evaluable for toxicity. Table 4 summarises drug-related haematological toxicities experienced by patients; the most common was neutropaenia in 83% (*n*=20) patients. Eighteen of the twenty-four (75%) patients experienced grade 3 or 4 neutropaenia, and in six of these, prolonged grade 4 neutropaenia constituted a DLT. Four patients developed febrile neutropaenia. Anaemia was significant (grade 3 or 4) in three patients. Grade 4 thrombocytopaenia was seen in one patient that was due to an idiopathic immune thrombocytopaenic purpura, with no clear relationship to study drug; the patient was on concomitant medication (quinine) that could have contributed to this. This thrombocytopaenia resolved with corticosteroid therapy. Overall, there was no evidence of cumulative myelosuppression with repeated dosing.

### Non-haematological toxicity

The most frequent drug-related non-haematological toxicities, occurring in ⩾25% of patients, are shown in Table 5. These comprised fatigue in 75% of patients, nausea in 58% and diarrhoea and vomiting in 46% of patients. Thirty-three per cent of patients experienced alopaecia and 25% dysgeusia. Constipation, cough and headache were seen in 17% of patients, each generally at grades 1–2 only (with 1 patient at dose level +1 experiencing grade 3 constipation). Peripheral neuropathy was mild and infrequent, being reported at grade 1 in five patients (two at dose level +1, one at dose level A and two at dose level A1) and grade 2 in two patients only (one each at dose levels 0 and A1). Mucositis was not reported. Overall, all toxicities were manageable, and there were no treatment-related deaths.

### Pharmacokinetics

Plasma concentrations from PK sampling were compared to plasma concentrations from phase I studies of ispinesib. A population PK analysis was conducted using NONMEM (Globomax LLC, Hanover, MD, USA) on phase I ispinesib data following an 18 mg m^−2^ dose, the MTD from a once every 21-day schedule (Chu *et al*, 2004). Using a validated population model, observed ispinesib concentration–time data from this study were overlaid on the simulated profile. Observed docetaxel data from subjects in this study administered 60 and 75 mg m^−2^ were overlaid with historical data from subjects dosed with 35, 75 and 100 mg m^−2^ docetaxel (Baker *et al*, 2003) to ascertain if an interaction was observed affecting docetaxel concentration–time profiles.

**Figure 1:**
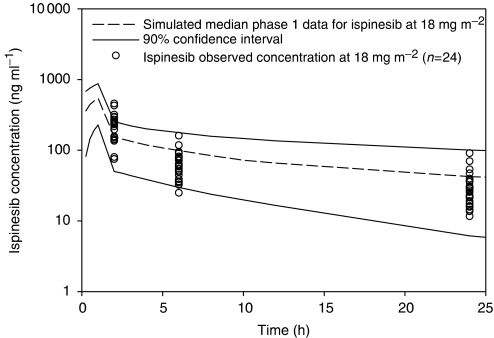
Simulated *vs* observed ispinesib concentration–time profiles at 18 mg m^−2^. Simulation of ispinesib concentrations based on population PK model developed from data collected in a single agent, first in human study of ispinesib in which subjects were dosed from 1–21 mg m^−2^.

**Figure 2:**
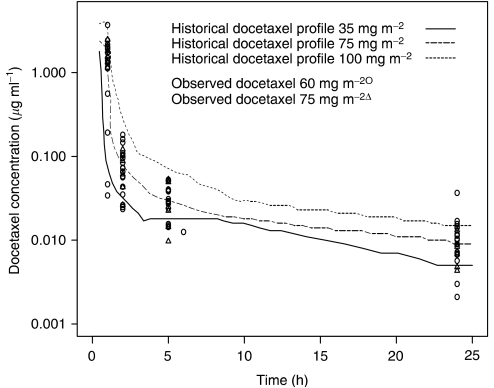
Observed and historical docetaxel concentration time profiles. Graph showing concentration time profiles of docetaxel for patients in this study (at 60 and 75 mg m^−2^) compared to historical controls (at 35, 75 and 100 mg m^−2^).

Using this model, ispinesib plasma concentrations in cycle 1 were consistent with those observed in phase I studies, as shown in Figure 1. Docetaxel PK parameters were consistent with those reported historically despite the co-administration of ispinesib (Figure 2; Baker *et al*, 2003).

### Response

There were no confirmed complete or partial responses. A total of seven patients had a best response of stable disease (SD) lasting ⩾18 weeks (six HRPC and one renal cell cancer), including one patient with HRPC demonstrating SD for ⩾24 weeks. Of the patients with HRPC, one demonstrated a confirmed >50% decrease in the serum level of prostatic-specific antigen (PSA).

## DISCUSSION

Antimitotic agents targeting tubulin, including the vinca alkaloids and taxanes, are arguably the most successful anticancer drugs developed to date. These findings have fuelled the development of novel antimitotics to improve drug disposition, decrease toxicity or improve efficacy. Recent drug discovery strategies have focused on the development of targeted agents that block the function of key enzymes involved in mitosis, such as the aurora kinases, polo-like kinase-1 (PLK-1) and the kinesins CENP-E and KSP (Blagden and de Bono, 2005; Jackson *et al*, 2007). These agents have shown promise in preclinical studies, and early clinical trial data indicate that they are well tolerated at biologically active doses, with neutropaenia being dose-limiting and showing little evidence of neurotoxicity. This improved toxicity profile may be advantageous; however, concerns remain about the basis for selective cytotoxicity with these agents. Moreover, owing to the neutropaenia associated with these drugs, combining these agents with established cytotoxics at recommended doses may be difficult.

In this phase I dose escalation study, ispinesib was combined with docetaxel. The drugs were administered consecutively on day 1 of a 21-day schedule and 24 patients were treated. No evidence of a drug–drug PK interaction was observed. The MTD for this study was defined as 10 mg m^−2^ of ispinesib and 60 mg m^−2^ of docetaxel. The tolerability profile was predictable, acceptable and manageable, with neutropaenia and leukopaenia occurring at a similar frequency to that seen with single agent docetaxel (Taxotere®, 2005). There was also sparing of the other haematopoietic lineages, which was also evident in phase I single agent studies of ispinesib (Chu *et al*, 2003, 2004; Burris *et al*, 2004). Peripheral neurotoxicity was generally mild and rarely seen, with grade 2 neuropathy observed in two patients receiving this regimen, supporting evidence that this novel targeted antimitotic is not by itself neurotoxic. Nonetheless, the cumulative overall administered dose of docetaxel in this study was low and may also partly explain the low rate of significant neurotoxicity.

The toxicity profile observed in this study was similar to that observed in preliminary reports of other phase I studies combining ispinesib with cytotoxic agents (Jones *et al*, 2006; Rodon *et al*, 2006). Rodon *et al* (2006) reported a DLT of grade 4 neutropaenia when ispinesib and capecitabine were administered on day 1 and days 1–14, respectively, of a 21-day schedule. However, Jones *et al* (2006) reported a DLT of grade 4 neutropaenia when ispinesib was combined with carboplatin on a day 1 every 21-day schedule.

The best objective tumour response observed was disease stabilisation lasting ⩾18 weeks in seven patients – six with HRPC and one with renal cell cancer. However, in this study, just one patient with HRPC demonstrated a confirmed ⩾50% decline in PSA (Bubley *et al*, 1999). This low PSA 50% decline rate in advanced HRPC patients may be attributed to the suboptimal dose of docetaxel administered, but it appears to suggest that KSP blockade does not increase the antitumour activity at the dose levels and schedule evaluated in this study.

Second generation KSP inhibitors are now in the clinic (Holen *et al*, 2006; Stein *et al*, 2006). SB-743921 is five-fold more potent than ispinesib against the ATPase activity of KSP and demonstrates cytotoxic activity at <2 nM in a range of tumour cell lines (Jackson *et al*, 2006). It has been tested in 44 solid cancer patients in a phase I study as a 1 h infusion administered every 21 days (Holen *et al*, 2006). The MTD has been defined as 4 mg m^−2^. MK-0731 has demonstrated an IC_50_ of 2.2 nM in several tumour cell lines and has been administered as a 24 h infusion every 21 days in eight patients so far (Stein *et al*, 2006). The MTD was exceeded at 48 mg m^−2^ and the recommended phase II dose is being explored. The preliminary reports of these two agents have shown a similar toxicity profile to ispinesib with DLTs of prolonged neutropaenia (Holen *et al*, 2006; Stein *et al*, 2006). The non-haematological toxicity profile of MK-0731 was similar to that of ispinesib with respect to gastrointestinal and constitutional toxicities; however, in contrast to our study and those of single agent ispinesib, mucositis, nail changes and phlebitis were reported (Stein *et al*, 2006). In addition, transaminitis, hyperbilirubinaemia and hypophosphataemia were reported with SB-743921 (Holen *et al*, 2006).

In conclusion, this study demonstrated that docetaxel can be safely administered with a KSP inhibitor but that non-cumulative neutropaenia limits the dosing of both these agents. Careful consideration needs to be given now to optimise the evaluation of the mitotic kinesin inhibitors through rational drug combinations that can lead to selective tumour cytotoxicity.

## Figures and Tables

**Table 1:** Planned dose escalation schema of ispinesib and docetaxel

	**Dose per 3 weekly cycle**	
**Cohort/dose level**	**Ispinesib**	**Docetaxel**
−2	6 mg m^−2^	50 mg m^−2^
−1	6 mg m^−2^	60 mg m^−2^
**0**	**8 mg m^−2^**	**60 mg m^−2^**
*A*	*10 mg m^−2^*	*60 mg m^−2^*
+1	8 mg m^−2^	75 mg m^−2^
*A*	*10 mg m^−2^*	*75 mg m* ^−*2*^
+2	12 mg m^−2^	75 mg m^−2^
+3	15 mg m^−2^	75 mg m^−2^
+4	18 mg m^−2^	75 mg m^−2^

Text in bold indicates the starting dose.

Text in italics indicates alternative dose levels that were planned if clarification of the MTD was required.

**Table 2:** Patient characteristics

Number of patients	24
Median age, years (range)	61.2 (41–76)
Gender, M/F	21/3
	
*Race*	
White	24
	
*ECOG PS*	
0	9
1	14
Unknown	1
	
*Tumour type*	
Prostate^a^	14
Duodenal adenocarcinoma	2
Cervix	2
Bladder	1
Renal^b^	2
Colon	1
Melanoma	1
Esophageal	1
	
*Previous treatment*	
Chemotherapy	8
Median number of previous chemotherapy regimens (range)	1.5 (1–3)
Radiotherapy	10
Surgery	9
Biological therapy	2

ECOG=Eastern Cooperative Group; F=female; M=male; PS=performance status

a All chemonaive.

b Previous immunotherapy.

**Table 3:** Dose levels, number of cycles administered and dose-limiting toxicities

	**Dose (mg m**^−**2**^)				
**Dose level**	**Ispinesib**	**Docetaxel**	***n***	**No. of cycles**	**DLT**
0	8	60	6	20	1 – prolonged grade 4 neutropaenia
+1	8	75	6	26	2 – prolonged grade 4 neutropaenia and febrile neutropaenia
A	10	60	3	12	0
A1	12	60	6	25	3^a^ – prolonged grade 4 neutropaenia
Ae	10	60	3	16	0

A=alternative dose level; Ae=expansion of cohort A at MTD; *n*=number of patients; No.=number.

a ⩾2 DLTs in cohort due to simultaneous enrolment of patients.

**Table 4:** Summary of drug-related haematological toxicities (all cycles)

**Toxicity**	**Grade**	**8/60^a^**	**8/75^a^**	**10/60^a^**	**12/60^a^**	**Total (%)**
Anaemia	1–2	—	3	2	1	6 (25)
	3–4	—	—	1	2	3 (13)
						
Neutropaenia	1–2	1	—	1	—	2 (8)
	3–4	4	5	4	5	18 (75)
						
Febrile neutropaenia	1–2	—	—	—	—	0 (0)
	3–4	—	2	—	2	4 (17)
						
Leukopaenia	1–2	—		1		1 (4)
	3–4	2	3	1	1	7 (29)
						
Thrombocytopaenia	1–2	—	—	2		2 (8)
	3–4	—	—	—	1	1 (4)
						

a Dose ispinesib/dose docetaxel (mg m^−2^).

**Table 5:** Summary of drug-related non-haematological toxicities in ⩾25% of patients

**Toxicity**	**Grade**	**8/60^a^**	**8/75^a^**	**10/60^a^**	**12/60^a^**	**Total (%)**
Lethargy/fatigue	1–2	4	5	5	3	17 (71)
	3–4	0	0	1	0	1 (4)
						
Nausea	1–2	3	0	5	5	13 (54)
	3–4	0	0	0	1	1 (4)
						
Vomiting	1–2	3	1	3	3	10 (42)
	3–4	0	0	0	1	1 (4)
						
Diarrhoea	1–2	1	1	4	2	8 (33)
	3–4	1	1	0	1	3 (13)
						
Alopaecia	1–2	2	2	2	2	8 (33)
	3–4	0	0	0	0	
						
Dysgeusia	1–2	1	1	2	2	6 (25)
	3–4	0	0	0	0	
						

a Dose ispinesib/dose docetaxel (mg m^−2^).
